# Context Matters: A Response to Autzen and Okasha’s Reply to Takacs and Bourrat

**DOI:** 10.1007/s13752-024-00455-7

**Published:** 2024-02-26

**Authors:** Peter Takacs, Pierrick Bourrat

**Affiliations:** 1 Department of Philosophy, The University of Sydney, Sydney, NSW Australia; 2 Department of Philosophy, Macquarie University, North Ryde, NSW Australia; 3 Charles Perkins Centre, The University of Sydney, Sydney, NSW Australia

**Keywords:** Evolutionary theory, Fitness, Growth rate, Philosophy of biology

## Abstract

In a recent reply to Takacs and Bourrat’s article (Biol Philos 37:12, 2022), Autzen and Okasha (Biol Philos 37:37, 2022) question our characterization of the relationship between the geometric mean and arithmetic mean measures of fitness. We here take issue with the claim that our characterization falls prey to the mistakes they highlight. Briefly revisiting what Takacs and Bourrat (Biol Philos 37:12, 2022) accomplished reveals that the key issue of difference concerns cases of deterministic but nonconstant growth. Restricting focus to such cases shows that there is in fact no reason for disagreement.

## Introduction

In a recent reply to Takacs and Bourrat’s “The Arithmetic Mean of What? A Cautionary Tale About the Use of the Geometric Mean as a Measure of Fitness” (2022), Autzen and Okasha (2022) question our characterization of the relationship between the geometric mean and arithmetic mean measures of fitness. The stated reason for their reply is that “[Takacs and Bourrat’s] assessment can lead to some mistaken ideas about the relationship between geometric mean fitness and population growth” (Autzen and Okasha 2022, p. 2). We share their desire for clarity and likewise wish to preempt any potential confusion about this vexing topic. However, several aspects of Autzen and Okasha’s response have left us somewhat bemused. In particular, we take issue with their claim that what we say, while apparently in one sense welcome, turns out to be either incorrect or correct for reasons other than the ones we have given. Their criticism thereby suggests that our characterization in fact falls prey to the mistakes they highlight rather than merely opening the door for misunderstanding. We respectfully disagree with this assessment. Briefly revisiting what Takacs and Bourrat (2022) accomplished reveals that the key issue of difference concerns cases of deterministic but nonconstant growth. Restricting focus to such cases, since those are the ones which feature most prominently in the philosophical literature on fitness,^1^ shows that there is no reason for disagreement.

## From Deterministic to Stochastic Settings and Back Again

The overarching aim in Takacs and Bourrat (2022) was to draw attention to the problems that can accompany claims about the geometric mean measure of fitness being *the* only reliable or perhaps even *the* best measure in deterministic, discrete-time settings like those that appear in influential philosophical works on the topic (Beatty and Finsen 1989; Brandon 1990; Sober 2001). As we illustrated, the instantaneous measure of fitness—the exponential of the arithmetic mean of the natural log growth rate (i.e., “Malthusian fitness”)—is just as good a measure as the geometric mean of offspring output when it comes to predicting relative evolutionary success in such cases. These are two ways of computing the exact same quantity (Crow and Kimura 1970, pp. 5–11). Some, though not all, prominent philosophers of biology have endorsed the geometric mean measure of fitness in discrete generation models without considering its problems. We accordingly explained why one might prefer Malthusian fitness on the grounds that it can often be applied in cases where a simple geometric mean measure like the one sometimes championed in the philosophical literature would not suffice.

Some might have read this as making an obvious point. We beg to differ. The notion of fitness customarily used by philosophers of biology relies primarily on reproductive output rather than growth rate. Notably, even Autzen and Okasha (2022) do not contest our diagnosis of this aspect *of the philosophical literature*. Although modellers and theoretical biologists may use the geometric mean and the intrinsic growth rate interchangeably, the links between these measures are rarely made explicit in the philosophical literature.

The cases used to illustrate our arguments were of two types. The first case (see Table 1 on page 6 in Takacs and Bourrat 2022) was inspired by a type of case that appears explicitly in Beatty and Finsen (1989) and Sober (2001). It involves a population of asexual conspecifics, each of whom exhibit one of two selectively relevant character states. We stipulated that the members of this population breed true to form, so the prospect of mutation was removed. Population growth was not density-regulated. There was no intragenerational variance. Type-A individuals contributed five offspring in odd-numbered years and six offspring in even-numbered years, whereas type-B individuals contributed two offspring in odd-numbered years and ten offspring in even-numbered years.^2^ Most important for present purposes was the fact that the reproductive schedules for these trait types were always of equal duration and over the same period. There was consequently no generational overlap.

The second case turned to population demography. In that example, we depicted two distinct human populations in place of two competing character states. The demographer’s interest in ascertaining which population grows faster accordingly supplanted the evolutionary biologist’s interest in trying to determine which character state would eventually prevail. A simplifying assumption in our demography example was that the populations were “internally uniform,” such that all individuals within a population were considered identical. The fitness of the population or the individuals that compose it is thereby equivalent. Each of these populations exhibited a unique discrete growth rate over unequal census times. The instantaneous growth rates that we subsequently calculated from the discrete growth rates were deterministic and constant. We assumed that progenitors always constitute a portion of subsequent population size, which eventually comes to include all descendants (i.e., offspring, grand-offspring, ad infinitum). Population growth was again independent of density. The purpose of this simple example from demography was to show the difficulties associated with assessing fitness (population growth rates) when reproductive schedules (census times) are not synchronized and, thereby, overlap. While using a greater number of populations would have made our point even stronger, restricting the example to just two allowed us to show exactly (mathematically) how one can use instantaneous growth rate to overcome the difficulties associated with assessing distinct growth rates over unequal census times when using the geometric mean. Our main point was that the instantaneous measure is *more pragmatically useful in an evolutionary setting than the simple geometric measure* because the (heritable) variation in reproductive timing might well be continuous in nature rather than restricted to just a few discrete “census times” (read “character states”).

The crucial point to note about both examples is that each assumes a form of deterministic growth. The first example includes deterministic but *nonconstant* growth rates for two trait variants, which makes (intergenerational) variance in reproductive output a critical factor for measuring relative evolutionary success. The second example invokes deterministic and *constant* growth rates for two distinct human populations. Transgenerational variance in growth rate was irrelevant in this latter case. In neither case were we addressing settings that involve nondeterministic or stochastic growth.^3^ With this clarification in hand, we can now proceed to show why the bulk of Autzen and Okasha’s criticism misses its intended mark.

Let us briefly restate their main argument before doing so. Autzen and Okasha begin by alleging that we have established a “false contrast” between the geometric and instantaneous measure (2022, p. 2): “[Takacs and Bourrat] go on to contrast geometric mean fitness with the instantaneous rate of natural increase *r* (or Malthusian parameter), which is defined as the exponential population growth rate on a continuous time scale.” Autzen and Okasha attempt to substantiate this allegation by noting that, “in continuous time models, which permit overlapping generations, there is in fact a quantity that is strictly analogous to the geometric mean fitness, known as the *long-run growth rate*, which determines the evolutionary outcome in a fluctuating environment” (Autzen and Okasha 2022, p. 2). The availability of this measure, they accordingly conclude, shows how “geometric mean fitness generalizes easily to the continuous setting, contrary to what Takacs and Bourrat imply” (Autzen and Okasha 2022, p. 2).

**Fig. 1:**
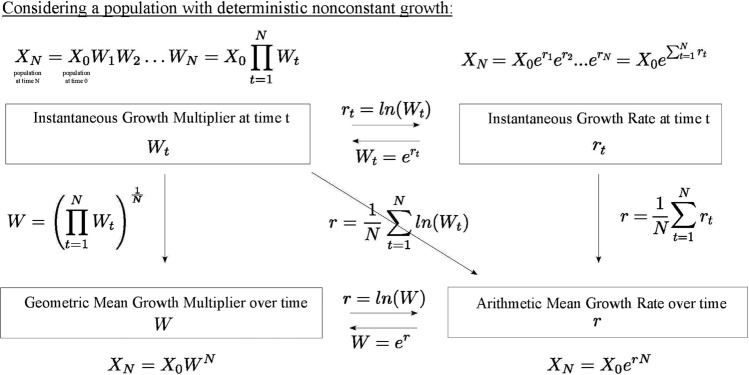
Relationship between the different measures used in Takacs and Bourrat (2022), Autzen and Okasha (2022), and the present article. Directional arrows and accompanying equations indicate the mathematical transformation of the source measure into the destination measure. Note that **r** in the figure would correspond to **s** in Autzen and Okasha (2022) if the model were stochastic. (See text for details)

This allegation is problematic for several reasons. First, even if the geometric mean as conceived of by many philosophers is a special case of the long-run growth rate (*s*) rather than what Autzen and Okasha choose to define as the Malthusian parameter (*r*),^4^ it is difficult to discern whether most philosophers of biology have in mind this more sophisticated measure (*s*) when contemplating geometric mean fitness. The toy examples of choice in the philosophical literature almost never introduce complex evolutionary scenarios and so do not require correspondingly sophisticated measures of fitness. Nevertheless, long-run growth rate (*s*) is a substantially different notion from the instantaneous growth multiplier (see *W*_*t*_ in Fig. 1), which is the simple geometric mean measure that most directly corresponds to that used in the philosophical literature. This much is already apparent in the terminology that Autzen and Okasha adopt. They describe the long-run growth rate (*s*) as being “strictly analogous” to geometric mean offspring output. By implication, it is not mathematically equivalent to the geometric mean measure for discrete-time settings (*W*_*t*_). It is designed to do similar work in a different (continuous) context. At a minimum, then, it must be an *extension of* the more basic geometric mean fitness function that appears in the once-influential philosophical literature on fitness.

Second and more substantially, it should be noted that their purported generalization of the Malthusian parameter (*r*) via *s* immediately presents a more complex model with additional parameters. This additional complexity is required for genuinely *stochastic* settings. The measure for long-term growth rate (*s*) assumes environmental stochasticity, infinite (or very large) population size, and accordingly the absence of within-generation (demographic) stochasticity. The Malthusian parameter as we used it (or the geometric mean fitness) provides us with a good way of modelling uncertainty when variation in fitness occurs “in a series,” such that the tokens of a genotype or phenotype have identical fitness within a generation but not necessarily across generations, as in the standard philosophical examples we targeted (Table 1 from Takacs and Bourrat 2022). Had we been interested in expounding on fitness measures for stochastic scenarios in addition to deterministic ones, we would have introduced more complex cases and used the appropriate fitness measures for those models. The important takeaway, though, was that there was no need to introduce this level of complexity or formal sophistication in order to demonstrate that the continuous time measure of fitness *r* is just as good as, if not in some cases better (for practical reasons) than, the basic geometric mean measure of offspring output for those cases featuring *deterministic but nonconstant* growth that appear in the philosophical literature.

Autzen and Okasha have inadvertently “shifted the goalposts” by extending consideration to stochastic settings. In nondeterministic settings, there are situations where long-term growth rate (*s*) and their preferred definition of the Malthusian parameter (*r*) may come apart. We agree that the long-term growth rate is an obvious candidate for fitness maximization in a nondeterministic context. We also share the belief that such settings present richer cases for the analysis of fitness functions. However, as we shall show in the following section, the Malthusian parameter (as defined in Takacs and Bourrat 2022) and long-term growth rate prove to be strictly equivalent measures of fitness in a deterministic setting.

## Long-Term Growth Rate and Its Relationship(s) with the Malthusian Parameter

Throughout Takacs and Bourrat (2022) we use the terms “intrinsic” (as in “intrinsic growth rate”), “instantaneous” (as in “instantaneous growth rate”), and “Malthusian” (as in “Malthusian fitness” or “Malthusian parameter”) interchangeably without distinction. Considering the aims of our paper and the simple models addressed, such synonymous usage was innocuous. However, it is only fair to point out that these notions might come apart in some contexts. Malthusian fitness could, for example, be understood as distinct from what some might call “the instantaneous growth rate.” When working with finite data sets consisting of numbers of individuals (e.g., offspring), one can transform what Takacs and Bourrat (2022, pp. 12–13) call the “finite rate of increase” over a discrete time step (*λ*_*t*_) in order to derive an instantaneous measure: ${\text{ln}}\left({\lambda }_{t}\right)={r}_{t}$. The finite rate of increase corresponds directly to what Autzen and Okasha call a “growth multiplier” (*W*_*t*_), which reflects mean fitness at time *t* in terms of numbers of offspring. The transformation from *W*_*t*_ (or *λ*_*t*_) into *r*_*t*_ implies a shift from manipulating numbers of individuals to the mathematical manipulation of log numbers of individuals. For clarity, we here denote such an instantaneous measure ‘*r*_*t*_’ in order to distinguish it from the instantaneous measure known as the Malthusian parameter *r* (without a subscript for time-indexing). The mathematical relationship between these two distinct instantaneous measures is shown in Fig. 1.

It is easy to see that the Malthusian parameter (*r*) is calculated as an arithmetic mean over the time-indexed instantaneous growth rates (*r*_*t*_) and is therefore equivalent to the arithmetic mean over log-transformed growth multipliers (ln(*W*_*t*_)).

Considering the potential for misunderstanding, it seems as though Autzen and Okasha may have misread what we meant by “Malthusian,” “instantaneous,” or “intrinsic” in one of two ways.

One possibility is that Autzen and Okasha read our usage of these terms as referring to *r*_*t*_ in contrast to the properly computed Malthusian *r*. Instantaneous fitness in the former (*r*_*t*_) sense refers to the exponential growth rate of a population measured over an infinitesimally short timestep (in continuous time) or with respect to a single timestep (in discrete time). From the perspective of an evolutionary biologist, this is usually not a very interesting measure because of its tendency to fluctuate dramatically as a result of demographic or environmental stochasticity. Few if any would condone its use for predicting or explaining differential evolutionary success. The importance of this measure lies primarily in that it provides values over which to average when calculating Malthusian fitness (*r*) as in Fig. 1, or what Autzen and Okasha would call “long-term growth rate” in a stochastic model (*s*). Note that the Malthusian parameter is the constant growth rate that would best approximate the growth rate of a population over many timesteps *even when growth is nonconstant*. So, unlike *r*_*t*_, it is obviously intended as *a long-term measure of fitness* and differential evolutionary success.

Another possibility for misunderstanding revolves around the meaning of a key term: “Malthusian.” The measure Autzen and Okasha choose (following Saether and Engen 2015) to label “Malthusian fitness” or “*r*” is not in fact the one that population biologists would normally associate with the term “Malthusian parameter” when applied in a stochastic context. Most would instead reserve this term for what Autzen and Okasha call “long-term growth rate” (*s*), defined as *s* = *E*(ln(*W*_*t*_)). What they opt to call the “Malthusian parameter (*r*)” in their response is defined in the following way: *r* = ln(*E*(*W*_*t*_)). Despite the occurrence of “*r*” as the *definiendum* here, this expected growth rate in fact has no commonly accepted name because nobody would use it for long-term projections of differential evolutionary success in a stochastic context. It has no equivalent in Fig. 1. Although wrong for long-term predictions of relative evolutionary success, this measure can still provide the safest bet *for short-term predictions of relative success*. Knowing nothing about growth in the previous generation, it provides us with a prediction of growth in the following generation that would most minimize the error associated with deviation of realized growth rate from expectation (Lewontin and Cohen 1969). If for no other reasons than the ones noted here, Autzen and Okasha should have foreseen that we would argue *for* rather than *against* the mathematical transformation of *W*_*t*_ into *r* (as on the diagonal in Fig. 1) and, therefore, the measure they call *s* when consideration shifts to a stochastic context.^5^

When it comes to cases involving deterministic nonconstant growth, the Malthusian parameter (“*r*” as we have defined it) turns out to be strictly equivalent to the long-term growth rate (*s*). The deterministic analogue of long-run growth rate (*s*) is depicted on the bottom left-hand side of Fig. 1 under the name “geometric mean growth multiplier” (over time). Since the calculation of fitness via the geometric mean growth multiplier (denoted here by *W* without time-indexing) obviously involves the geometric mean, it is this measure (*W*) that Autzen and Okasha should claim as “strictly analogous” to the discrete-time geometric mean measure of fitness that appears in the prominent philosophical literature (Beatty and Finsen 1989; Brandon 1990; Sober 2001). The central point to note is that *W* is mathematically equal to the exponential of the Malthusian parameter (see bottom portion of Fig. 1). In Takacs and Bourrat (2022), we sought to show why the instantaneous growth multiplier *W*_*t*_ (as adopted in acclaimed philosophical work) does not always suffice as a measure of fitness by examining instances where the Malthusian parameter becomes necessary. We accordingly rehearsed the well-worn, diagonal move from the upper left-hand side to the lower right-hand side of Fig. 1. Autzen and Okasha read this as foreclosing the possibility that there can be an adequate, “strictly analogous” geometric mean measure of fitness for continuous-time settings. Never did we preclude such a possibility, and for good reason. We could only have done so on pain of contradiction, as Fig. 1 makes abundantly clear. If the deterministic analogue (*W*) of long-term growth rate (*s*) is indeed the quantity that natural selection aims to maximize, then so, too, must selection seek to maximize *r*. For these are mathematically interdefinable measures of one and the same quantity (i.e., *W* = *e*^*r*^, *r* = ln(*W*)).

What, then, ultimately accounts for Autzen and Okasha’s disagreement with us? In large part it is just the fact that they apply what we say in Takacs and Bourrat (2022) to nondeterministic (stochastic) cases. Had we in fact adopted their preferred definition of *r* from a stochastic context (see Saether and Engen 2015), then our claims about geometric mean offspring output as a measure of fitness being a “special case of” the Malthusian parameter would have been incoherent. But we purposely restricted ourselves to cases that involve deterministic growth. Why? For starters, these are the types of cases that have traditionally dominated the philosophical landscape. Second and no less importantly, using such very simple cases was by far the most efficient way to convey our main message regarding the move away from a longstanding fixation on reproductive output toward growth rate. Autzen and Okasha’s reply should thus be read as a carefully considered “extension” (or perhaps “generalization”) of our claims to stochastic settings, which quite candidly present more interesting cases for fitness measurement. We already noted in Takacs and Bourrat (2022, p. 15) how open we are to such a possibility.

## Conclusion

Insofar as Autzen and Okasha’s critical reply preempts future confusion surrounding the measurement of fitness, we welcome their contribution even if that hoped-for clarity comes partially at our expense. However, Takacs and Bourrat (2022) does not in fact make the mistake(s) they allege. The argument that we have erred depends crucially on an unfounded assumption, namely that the claims made in Takacs and Bourrat (2022) were supposed to hold in stochastic contexts. As we have shown, this was not the case. Our arguments were intended only for deterministic contexts, which notably admit of both constant and nonconstant growth. In deterministic settings, long-term growth rate (*s*) collapses into the Malthusian parameter *r* (as we define it).This is evinced by the fact (see Fig. 1) that the continuous analogue of the discrete geometric mean measure of offspring output in a deterministic setting—the geometric mean growth multiplier (over time)—is mathematically equivalent to the Malthusian parameter. There is consequently no compelling reason for disagreement on this front. We gladly embrace what Autzen and Okasha say about fitness measures for stochastic contexts.

We believe that Autzen and Okasha’s critical reply was penned with the commendable intention of eradicating what they see as a misguided reflex in those who engage with the modelling literature in population biology. The broad target of their questioning is the presumption that there must be a “uniquely correct” measure of fitness that is somehow free from modelling assumptions. It is in this specific sense that they apparently recoil at our “championing” the arithmetic measure of fitness. They contend that “the evidence strongly suggests that there is no such definition; and evolutionary biology seems to get on fine without one” (Autzen and Okasha 2022, p. 10). But the fact that many biologists may never take the time to open their theoretical closets does not imply that there are no telling philosophical remnants hiding therein. While we offer no definitive answer to their contention, the metaphysical agnosticism implied by their view strikes us as having a number of problematic consequences for evolutionary biology (Takacs and Bourrat 2021). Our claim in Takacs and Bourrat (2022) is that the long-term arithmetic mean growth rate (Malthusian parameter, as we here define it) has proven to be the most general measure of fitness in terms of its unparalleled applicability. In light of what we have now shown, Autzen and Okasha should agree with this point since long-run growth rate (*s*) is either (1) equivalent to the Malthusian parameter/fitness in a deterministic context or (2) precisely the function that is picked out by the term “Malthusian parameter/fitness” in a stochastic setting. Predictive efficacy is inevitably a comparative notion, not an absolute one. The arithmetic mean measure of fitness that we advocate seems to be the “best of the bunch” when assessment is restricted to the range of cases that currently interest evolutionary population biologists. This relative and thus very much attenuated form of supremacy, when understood as a properly epistemic (theoretical) virtue, is reason enough to “champion” the arithmetic measure for the time being.

Matters would, of course, become much more contentious if we were to draw the conclusion that our preferred measure of fitness more accurately reflects whatever it is in nature that corresponds to the fodder for selection. Yet even this treacherous transition from epistemology to ontology, while perhaps considered passé in some circles, is not without precedent. One can be a scientific realist as well as a fallibilist by maintaining that “realism is appropriate in connection with our best theories even though they likely cannot be proven with absolute certainty” (Chakravartty 2017). Variations in this vein include those who advocate for “internal realism” (Putnam 1981; Ellis 1988) or “semirealism” (Chakravartty 1998). Now is not the time for a lengthy discussion of this familiar and highly contentious issue. We raise this point merely to note that it is not obviously unreasonable to assume that the “best” measure (in terms of range of application) that we currently have for a fundamental feature of our consensus explanation of adaptive evolution might also extend beyond our collected heads. Shifting from a geometric mean measure to a measure of fitness based on growth rate changes the way we think about the basic currency of selection. In particular, it refocuses our attention on the phenomenon of (relative) growth unencumbered by a restriction to number of offspring (Van Valen 1976, 1989; Bouchard 2008, 2011; Takacs and Bourrat 2022, pp. 18–19). That a theory happens to be a construct consisting of models and logical relations can sometimes obscure the fact that it is ultimately designed to explain the phenomena *in the world* that inspire its construction. Theories, even when sensibly construed as “patchworks of models” (sensu Cartwright 2008), must at some point confront the “tribunal of experience” (Quine 1980).

## Data Availability

Not applicable.

## Footnotes

1 There are notable exceptions to this trend (see especially Autzen 2020).

2 On page 5 of Takacs and Bourrat (2022), we stated the following: “Type-A individuals can contribute either five or six offspring *with equal probability*, while type-B individuals can contribute either two or ten offspring *with equal probability*” (italics added here for emphasis). This was a misleading articulation of the case, for it suggests that the model is stochastic rather than deterministic. In our periodic deterministic model, we know that, if the current year is a “good” year in terms of growth, the next year will necessarily be a “bad” one and vice versa. These growth multipliers (i.e., “single generation growth rates” in Takacs and Bourrat 2022) are thus *not* independent and identically distributed (“i.i.d.”).

3 See footnote 11 in Takacs and Bourrat (2022) for a candid statement on this issue.

4 Throughout our response readers will come across qualifications such as “what they choose to define as” or “their preferred definition of” with respect to the Malthusian parameter. The reason for such qualification will become apparent in the following section.

5 Autzen and Okasha’s reading of Takacs and Bourrat (2022) as adopting their preferred definition of *r* (i.e., *r* = *ln*(*E*(*W*_*t*_)), from Saether and Engen 2015) is somewhat perplexing given the context of our work more generally. A unifying theme throughout much of our work is that the proper characterization of evolutionary processes requires scrutiny of temporal scale (e.g., Bourrat 2015; Black et al. 2020; Doulcier et al. 2021; Takacs and Bourrat 2021).
